# Supplementary material for: Impact of Liver Metastases and Number of Metastatic Sites on Immune-Checkpoint Inhibitors Efficacy in Patients with Different Solid Tumors: A Retrospective Study
Source: Biomedicines. 2022 Dec 29;11(1):83. doi: 10.3390/biomedicines11010083 (PMC9855949; doi:10.3390/biomedicines11010083)
Supplement: Supplementary file 1 [file biomedicines-11-00083-s001.zip › biomedicines-1803010-supplementary.pdf]

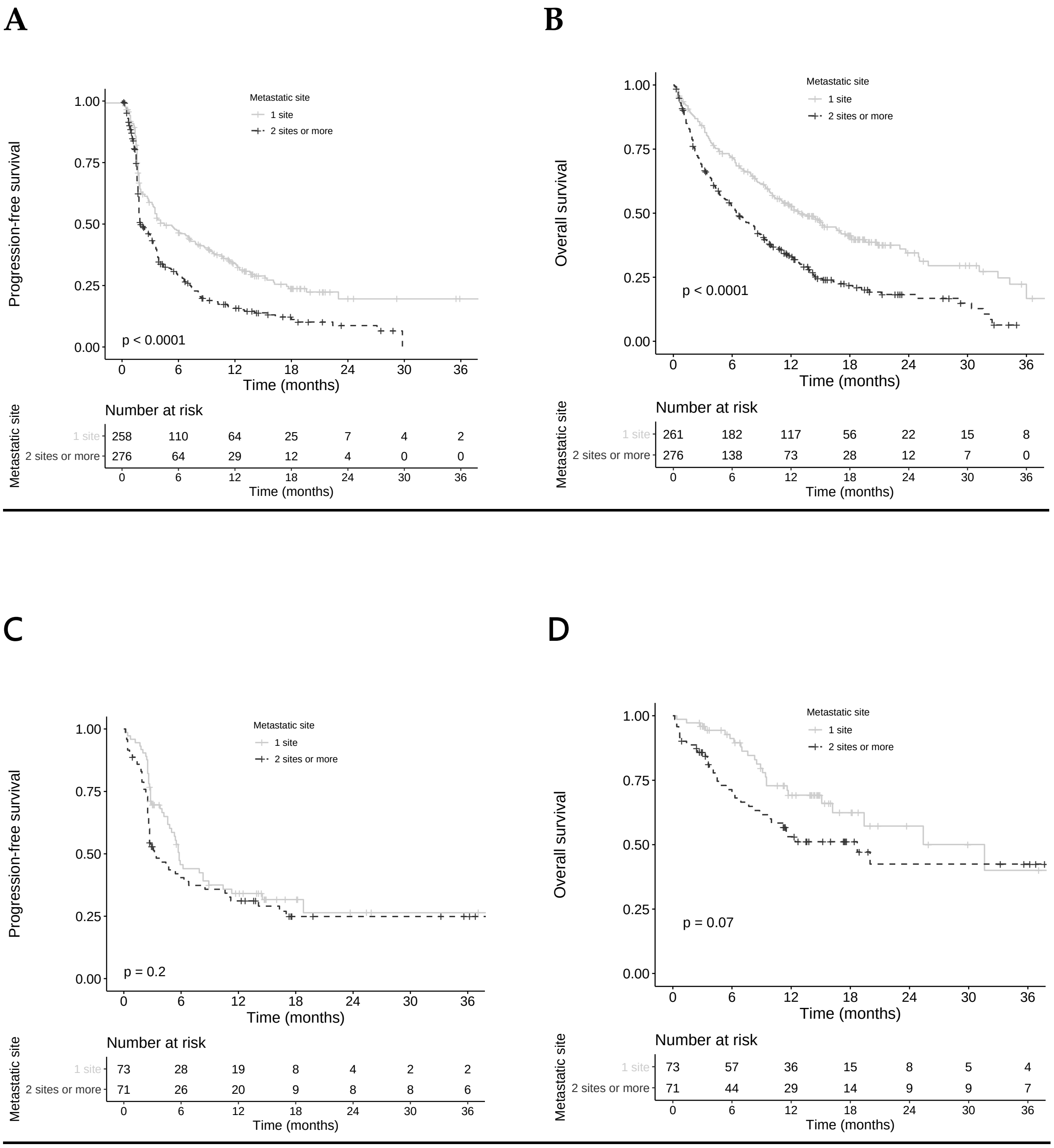

Figure S1: Kaplan-Meyer analyses. (A) Progression-free survival (PFS) according to metastatic site (MS) number (1 MS versus ≥ 2 ML) in patients with metastatic NSCLC (B) Overall survival according to MS number (1 MS versus ≥ 2 ML) in patients with metastatic NSCLC. (C) Progression-free survival (PFS) according to metastatic site (MS) number (1 MS versus ≥ 2 ML) in patients with metastatic melanoma. (D) Overall survival according to MS number (1 MS versus ≥ 2 ML) in patients with metastatic melanoma.

A

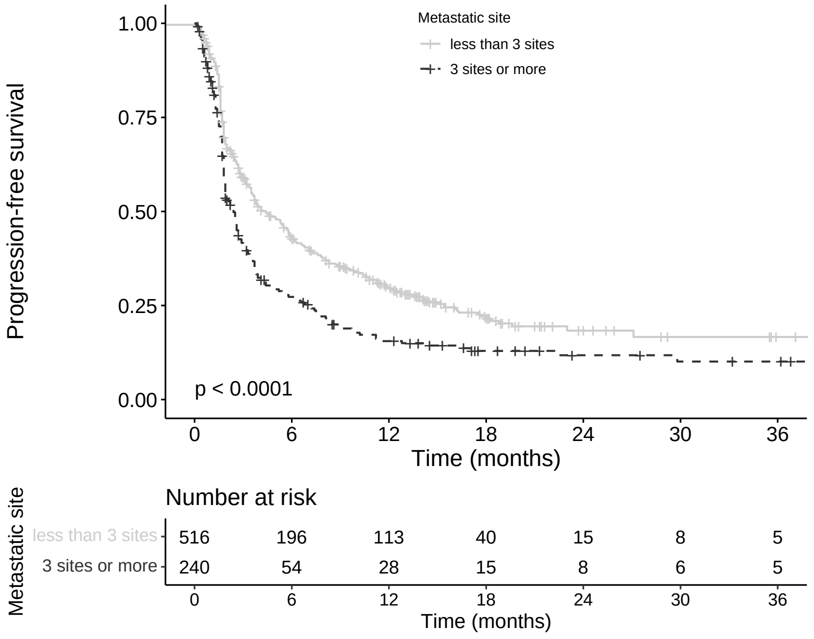

B

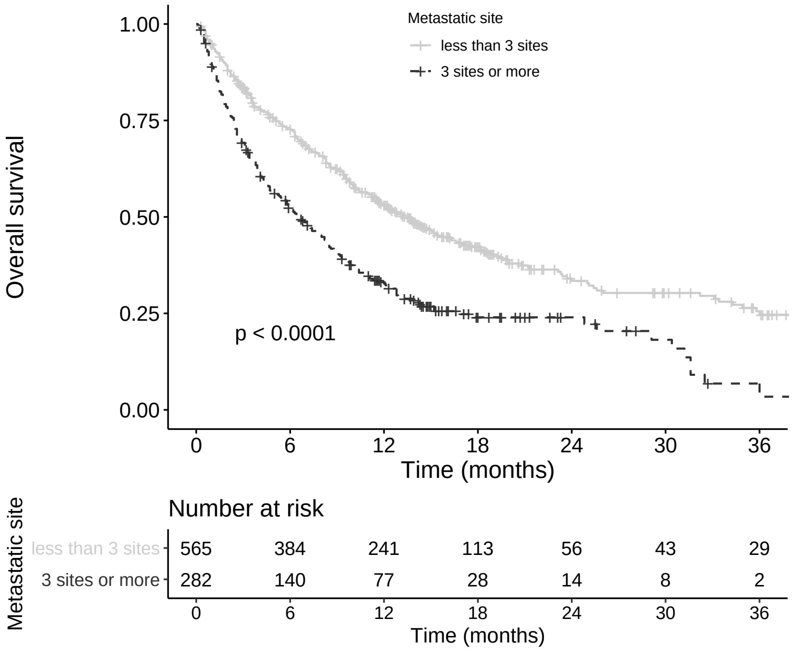

Figure S2: Kaplan-Meyer analyses. (A) Progression-free survival (PFS) according to metastatic site (MS) number (< 3 MS versus ≥ 3 ML) (B) Overall survival according to MS number (< 3 MS versus ≥ 3 MS).

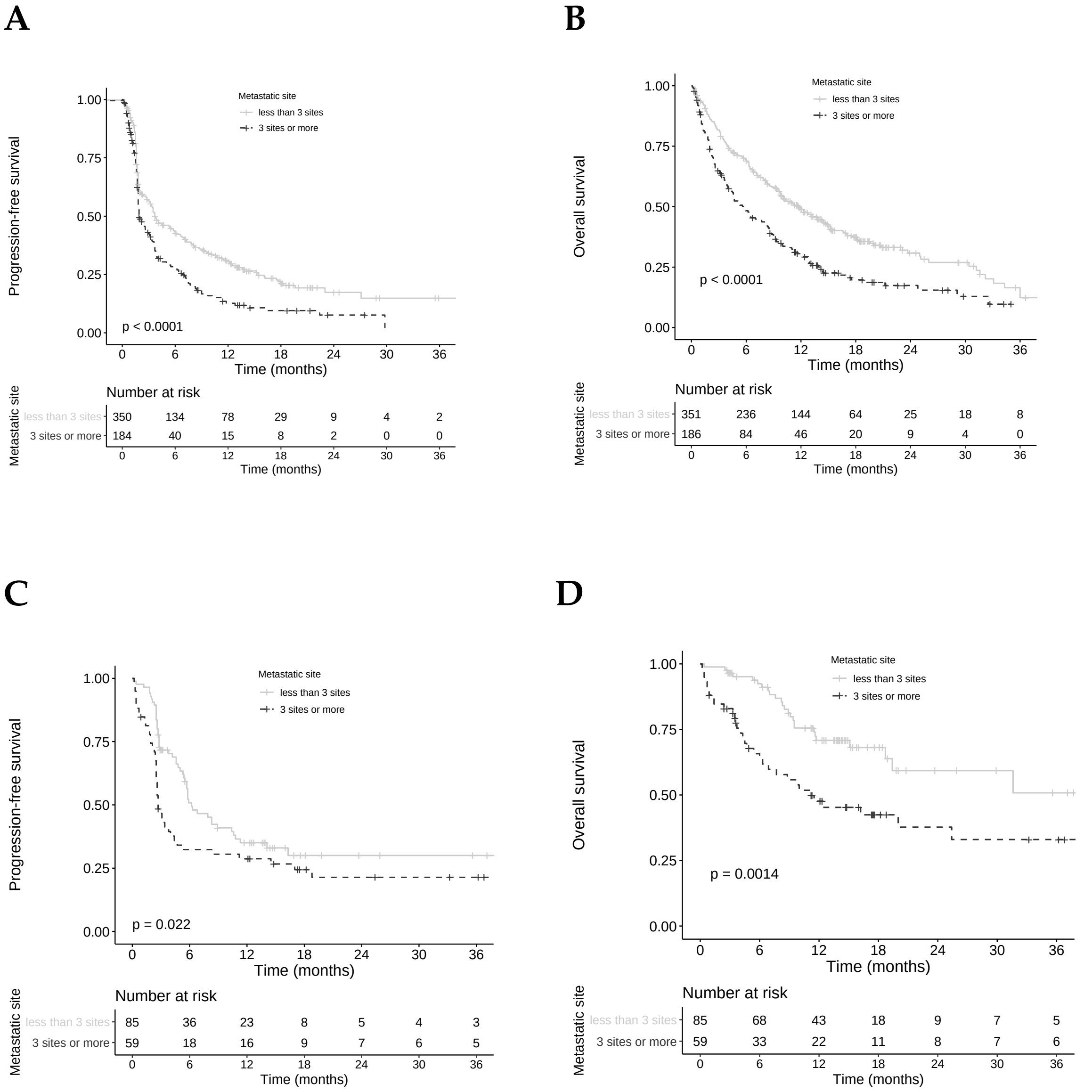

Figure S3: Kaplan-Meyer analyses. (A) Progression-free survival (PFS) according to metastatic site (MS) number (< 3 MS versus ≥ 3 MS) in patients with metastatic NSCLC (B) Overall survival according to MS number (< 3 MS versus ≥ 3 MS) in patients with metastatic NSCLC. (C) Progression-free survival (PFS) according to metastatic site (MS) number (< 3 MS versus ≥ 3 MS) in patients with metastatic melanoma. (D) Overall survival according to MS number (< 3 MS versus ≥ 3 MS) in patients with metastatic melanoma.

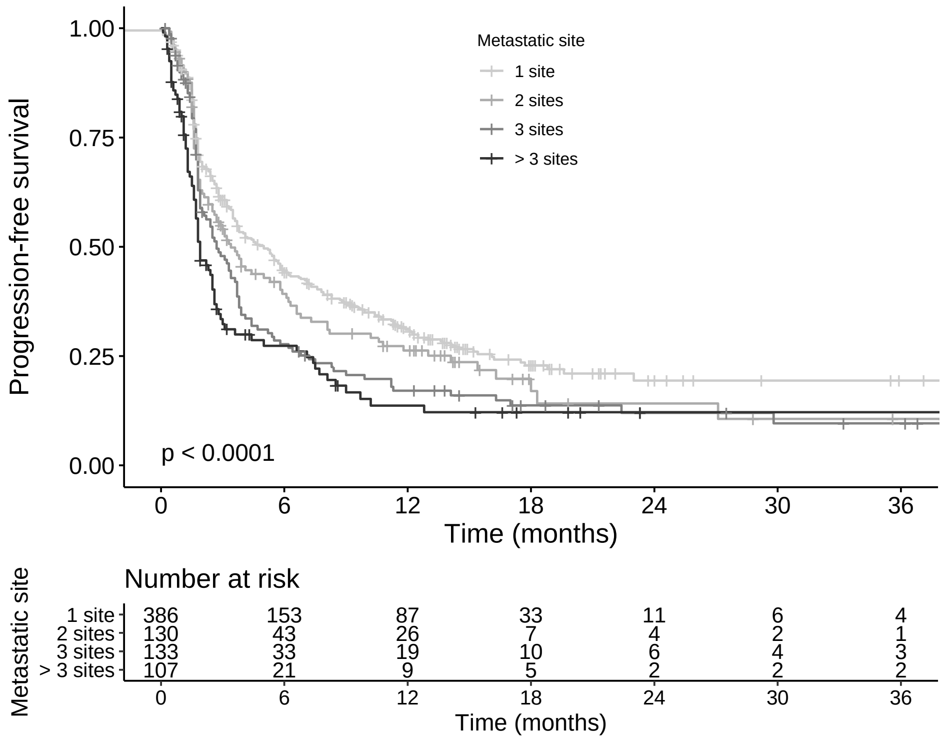

Figure S4: Kaplan-Meyer analyses. Progression-free survival (PFS) according to metastatic site (MS) number (1MS versus 2MS versus 3MS versus >3MS).

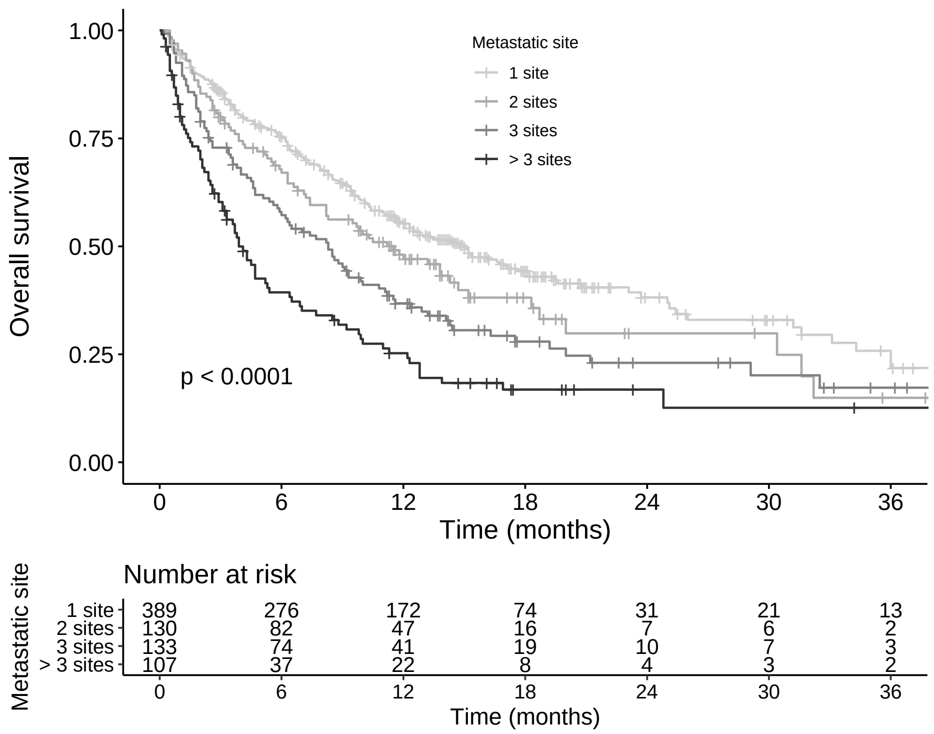

Figure S5: Kaplan-Meyer analyses. Overall survival according to metastatic site (MS) number (1MS versus 2MS versus 3MS versus >3MS).

Table S1. Progression-free survival prognostic factors in univariate and multivariate analysis (Cox model)

Characteristics	N (%)	Progression-free survival				
			Unadjusted analysis		Adjusted Analysis	
		Median PFS (95% CI)	HR (95%CI)	P	HR (95%CI)	P
**Age NA=0**						
**< 70**	489 (64%)	3.2 (2.7-3.7)	REF	0.11	REF	0.11
**≥70**	270 (36%)	4.1 (3.2-5.5)	0.87 (0.72-1.0)		1.6 (0.9-3.0)	
**PS < 2** ***(%) NA=33***	534 (74%)	4.4 (3.7-5.6)	0.55 (0.45-0.67)	<0.0001	0.59 (0.48-0.71)	<0.0001
**PS ≥ 2**	192 (26%)	1.9 (1.8-2.7)	REF		REF	
**Primary tumor** ***(%) NA=0***						
**NSLC**	537 (71%)	3.3 (2.6-3.7)	REF	0.0014	REF	0.0096
**Melanoma**	144 (19%)	5.4 (3.8-7.9)	0.69 (0.55-0.87)		0.73 (0.58-0.93	
**Urologic**	78 (10%)	3.2 (2.3-5.0)	1.16 (0.89-1.51)		1.24 (0.95-1.64)	
**Nb of metastatic site**						
**1**	389 (51%)	5.0 (3.7-6.0)	REF	<0.0001	REF	0.061
**>1**	370 (49%)	2.6 (2.3-3.2)	1.41 (1.19-1.67)		1.19 (0.99-1.44)	
**Liver metastasis**						
**No**	592 (78%)	4.0 (3.6-5.4)	REF	<0.0001	REF	<0.0001
**Yes**	167 (22%)	1.9 (1.8-2.5)	1.63 (1.34-1.98)		1.57 (1.27-1.96)	
